# Knowledge, attitude, and practice of nurses concerning immediate actions during vehicle drowning

**Published:** 2022-12

**Authors:** Fatemeh Mansouri, Azar Darvishpour, Shiva Mahdavi Fashtami

**Affiliations:** ^*a*^ Zeynab (P.B.U.H) School of Nursing and Midwifery, Guilan University of Medical Sciences, Rasht, Iran.; ^*b*^ Social Determinants of Health (SDH) Research Center, Guilan University of Medical Sciences, Rasht, Iran.; ^*c*^ Pirouz Hospital, Guilan University of Medical Sciences, Rasht, Iran.

## Abstract

**Background:**

Car drowning has one of the highest mortality rates among all types of motor vehicle accidents. Many of these drownings can most likely be avoided if the occupants know what to do and act quickly. Having awareness in dealing with such situations seems necessary for all sectors of society, especially health workers. The purpose of this study was to assess the knowledge, attitudes, and practice of nurses concerning immediate action during the immersion of vehicles in the water.

**Methods:**

This descriptive study was conducted in 2022 as an online survey. Ninety-five nurses were selected and completed the KAP questionnaire (knowledge, attitude, and practice) according to the instructions of ALIVE: (Automobile Submersion: Lessons In Vehicle Escape). Descriptive statistics were used to analyze the data using an online questionnaire system (Porsline).

**Results:**

The majority of the subjects (94.7%) were female and in the age group of 45-36 years (41%). Most of them had a bachelor's degree (72.6%) and a work experience of 10-15 years (25.2%). The majority of respondents (74.7%) stated that they had not received any information about the vehicle sinking from various media. Most (78.9%) of them were not aware of the recommended protocol for rescuing themselves when the vehicle sank. Thirty-four percent of nurses felt that their risk of getting caught in vehicle immersion was high and the probability of their survival was average (50%). Of all respondents, 82% chose "a successful initial attempt to escape". Other responses indicated that they were less likely to complete a successful process to save themselves.

**Conclusions:**

According to the results, we should focus on the following points: passengers only have about 1 minute to get out of a sinking car and they should not rely on emergency contact but should follow the SWOC protocol (“SEATBELTS” off, “WINDOWS” open, “OUT” immediately, “CHILDREN” first.

**Keywords:**

Drowning, Vehicles, Nurse

